# Results of a Randomized, Open-Label, Clinical Trial Investigating the Effects of Supplementation with *Heracleum persicum* Extract as an Adjunctive Therapy for Dyslipidemia

**DOI:** 10.1100/tsw.2011.43

**Published:** 2011-03-07

**Authors:** Yunes Panahi, Bahram Pishgoo, Fatemeh Beiraghdar, Zahra Mohammadi Araghi, Amirhossein Sahebkar, Ehsan Abolhasani

**Affiliations:** ^1^ Chemical Injuries Research Center, Baqiyatallah University of Medical Sciences, Tehran, Iran; ^2^ Department of Cardiology, Baqiyatallah University of Medical Sciences, Tehran, Iran; ^3^ Nephrology and Urology Research Center, Baqiyatallah University of Medical Sciences, Tehran, Iran; ^4^ Pharmaceutical Sciences Branch, Islamic Azad University, Tehran, Iran; ^5^ Cardiovascular Research Center, Avicenna Research Institute, Mashhad University of Medical Sciences (MUMS), Mashhad, Iran; ^6^ Biotechnology Research Center and School of Pharmacy, Mashhad University of Medical Sciences (MUMS), Mashhad, Iran; ^7^ Medicine Faculty, Shaheed Beheshti University of Medical Sciences, Tehran, Iran

**Keywords:** Heracleum persicum, atorvastatin, dyslipidemia, cholesterol, triglycerides

## Abstract

The present study evaluated the potential benefit of supplementation with *Heracleum persicum* as an adjunctive therapy to atorvastatin in dyslipidemic subjects. In a randomized, open-label, clinical trial, 100 dyslipidemic subjects were randomly assigned to: (1) *H. persicum* group (*n* = 50, completers = 18), receiving *H. persicum* extract (500 mg/day) + atorvastatin (10 mg/day) for 8 weeks, or (2) atorvastatin group (*n* =50, completers= 34), receiving only atorvastatin (20 mg/day) for 8 weeks. Weight, body mass index (BMI), lipid profile, and biomarkers of hepatic and renal injury were determined at baseline and at the end of the trial. There were significant reductions in serum total cholesterol and LDL-C in both the *H. persicum* (*p* = 0.001) and atorvastatin (*p* < 0.05) groups. Serum HDL-C was elevated in the atorvastatin group (*p* < 0.05), while no significant change was observed in the *H. persicum* group (*p* > 0.05). Serum triglyceride levels remained statistically unchanged by the end of the trial in both groups (*p* > 0.05). Serum alanine (*p* = 0.049) and aspartate aminotransferase (*p* = 0.013) levels rose in the atorvastatin, but not the *H. persicum* (*p* > 0.05) group. In comparison with baseline values, no significant change was observed in weight and BMI, as well as serum levels of creatinine, blood urea nitrogen, and fasting blood sugar in either of the groups (*p* > 0.05). Apart from HDL-C, the effects of atorvastatin (20 mg/day) on other lipid profile parameters do not appear to be significantly superior to those achieved by combination therapy with *H. persicum* + atorvastatin (10 mg/day).

## INTRODUCTION

Coronary artery disease (CAD) is the most common cause of mortality and a major cause of disability worldwide, accounting for about 500,000 deaths annually in the U.S.[1]. Given the pivotal role of atherosclerosis as the underlying cause of CAD, controlling the risk factors of this focal inflammatory and fibroproliferative disease of arterial intima[2] would lead to a great increase in survival rate, as well as reduction of CAD incidence and consequent acute coronary events.

Dyslipidemia is widely regarded as one of the primary and major risk factors for atherosclerosis and CAD, and may even be considered as a prerequisite for CAD. However, in spite of its significant contribution to the development and progression of CAD, dyslipidemia is among the modifiable cardiovascular risk factors. Over the past decade, the inhibitors of cholesterol biosynthesis known as statins (which act by blocking the 3-hydroxy-3-methyl-glutaryl coenzyme A [HMG-COA] reductase enzyme) have been the most popular drug class for dyslipidemia. Generally, statins have been shown to be effective in both primary and secondary prevention of cardiovascular disease as well as decreasing the incidence of coronary events and cardiovascular mortality[3,4,5,6]. Recent data have indicated that clinical efficacy of statin therapy may not be completely attributed to their cholesterol-lowering actions, as these drugs possess several cholesterol-independent or "pleiotropic" effects, such as antioxidant, anti-inflammatory, and immunomodulatory properties, which play a role in their observed clinical benefits[7,8]. However, while statins are generally considered as safe drugs, there are some concerns regarding their side effects, such as muscular and hepatic complications, which become more important when noting the increasing trend to statin consumption and relatively prolonged treatment period with these drugs. As the side effects of statins seem be dose related, adjunctive therapy with effective and safe hypolipidemic agents may be an alternative approach in order to reduce the dose and side effects. To achieve such an adjunctive therapy, herbal medicine is a promising option.

*Heracleum persicum* (Persian hogweed) is a large and flowering shrub that belongs to the family Apiaceae. This perennial plant is endemic to Iran and is commonly known as "golpar" in Persian. Aromatic fruits of *H. persicum* are widely used in the daily diet of the Iranian general population as a flavoring agent. In addition, these fruits are used in Iranian traditional medicine as a carminative, digestive aid, antimicrobial tonic, antiepileptic, and aphrodisiac[9,10,11,12,13]. Modern research has also unveiled some other biological activities of *H. persicum,* such as immunomodulatory[14], anti-inflammatory[15], analgesic[15], anticonvulsant[16], cytotoxic[17], and antioxidant[18] properties. However, clinical investigations on this plant are lacking, with no previous scientific report on its possible cardioprotective or hypolipidemic activity. Regarding the introduction of several interesting medicinal herbs and phytochemicals with lipid-lowering actions (e.g., garlic, green tea, soy products, red yeast, plant stanols, etc.)[19], the present study aimed to evaluate the potential benefit of *H. persicum* supplementation as an adjunctive therapy to atorvastatin in dyslipidemic subjects.

## METHODS

### Subjects and Design

This was a randomized, open-label, clinical trial that was performed between June 2009 and June 2010 in the Cardiovascular Clinic of Baqiyatallah Hospital (Tehran, Iran). Men and women between 35 and 80 years of age who were not taking any lipid-lowering drug during the past 2 months and had at least one of the following criteria were included in the study: (1) total cholesterol >200 mg/dL; (2) LDL-C >130 mg/dL; and (3) HDL-C <35 mg/dL. Exclusion criteria were the presence of an acute coronary syndrome; history of angioplasty or by-pass surgery; thyroid, hepatic, or renal (including nephrotic syndrome) abnormalities; pregnancy; lactation; intolerance to the prescribed medication for any reason; history of hypersensitivity to HMG-COA reductase inhibitors; and concurrent use of medications that might affect on plasma lipids.

One hundred subjects met the eligibility criteria and were randomly assigned to: (1) *H. persicum* group ($n$ = 50), receiving dried *H. persicum* extract (500 mg/day) + atorvastatin (10 mg/day) for 8 weeks, or (2) atorvastatin group ($n$ = 50), receiving only atorvastatin (20 mg/day) for 8 weeks. Randomization was performed individually and patients were alternatively allocated to capsules coded A or B with the first code being chosen randomly.

*H. persicum* extract used in the study was in the form of Lipicom® capsules (Barij Esssence Pharmaceutical, Mashade Ardehal, Kashan, Iran), each containing 250 mg of dried plant extract.

Anthropometric parameters including weight, height, and body mass index (BMI), together with serum lipid profile (including total cholesterol, triglycerides, LDL-C, and HDL-C) and levels of aspartate aminotransferase (AST), alanine aminotransferase (ALT), alkaline phosphatase (ALP), fasting blood sugar (FBS), creatinine, and blood urea nitrogen (BUN), were measured for participants at baseline and at the end of the study using routine laboratory tests. LDL-C was directly measured using a commercial kit. In addition, information regarding self or family history of diabetes mellitus, hypertension, angina pectoris, stroke, and cardiovascular disease hospitalization; previous consumption of lipid-lowering drugs; smoking habit; and drug side effects were gathered using a questionnaire at baseline and at the end of the study.

The study protocol was approved by the institutional Ethics Committee and written informed consent was obtained from participants.

## Statistical Analysis

Sample size calculation ($n$ = 50 in each group) was performed with an estimated power of 90% and $\alpha$ = 0.05 to detect a 25% decrease in serum triglycerides.

Statistical analyses were performed using SPSS 17 for Windows software. Values were presented as frequency (%) or mean ± SD. Between- and within-group comparisons were performed using the Mann-Whitney U test and Wilcoxon signed ranks test, respectively. Categorical variables were compared using Chi-square or Fisher's Exact test.

## RESULTS

From the 100 patients who were evaluated for the trial, 52 completed the study ($n$ = 18 and 34 in the *H. persicum* and atorvastatin groups, respectively) and were included in the final analysis (Fig. 1). As for the drop outs, the main reason was loss to follow-up rather than lack of compliance, and loss to follow-up was due to not keeping the appointment for the last visit to obtain a post-trial blood sample. A small number of drop outs was due to the lack of compliance based on the self-report.

## Demographic Data

The two groups were not significantly different at baseline regarding gender, weight, BMI, serum lipid profile parameters (comprising total cholesterol, triglycerides, LDL-C, and HDL-C), transaminases (ALT and AST), and ALP ($p > 0.05$). However, the atorvastatin group was significantly older than the *H. persicum* group ($p < 0.05$). Serum levels of FBS ($p < 0.001$) and BUN ($p < 0.001$) were significantly higher in the atorvastatin group, while serum creatinine was significantly higher in the *H. persicum* group ($p < 0.05$). The prevalence of present or past hypertension, angina pectoris, stroke, hospitalization due to cardiovascular diseases, and renal and hepatic disorders was not significantly different between the groups ($p > 0.05$), but there was a higher prevalence of diabetes mellitus in the atorvastatin group ($p < 0.01$). As for the family history, the groups were not significantly different in the prevalence of hypertension and cardiovascular mortality ($p > 0.05$), whereas diabetes mellitus was more prevalent in the

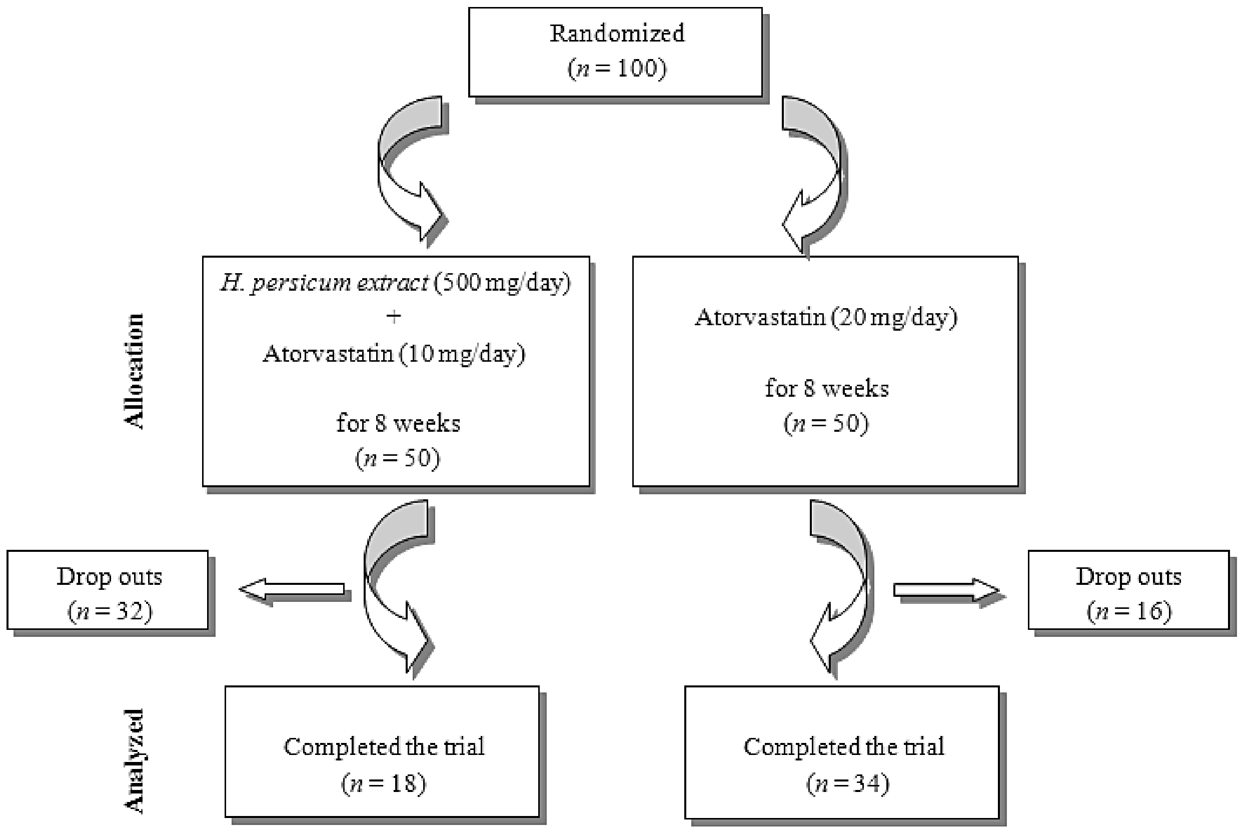

**FIGURE 1.** Flowchart of the trial.

atorvastatin group ($p < 0.01$, Tables 1 and 2). Patients were asked for their diet at baseline and at the end of the trial. Available records are summarized in Tables 3 and 4.

## Effect of Treatments on Weight, BMI, and FBS

In comparison to the baseline values, there was no significant change regarding weight, BMI, and FBS, neither in the *H. persicum* nor in the atorvastatin group ($p > 0.05$, Table 1). The magnitude of changes in weight, BMI, and FBS were not significantly different between the groups either ($p < 0.05$, Table 5).

## Effect of Treatments on Serum Lipid Profile Parameters

There were significant reductions in serum total cholesterol and LDL-C in both the *H. persicum* ($p = 0.001$) and atorvastatin groups ($p < 0.001$). Serum HDL-C was elevated by the end of the trial in the atorvastatin group ($p < 0.05$), but no significant change was observed in the *H. persicum* group ($p > 0.05$). Serum triglyceride levels remained statistically unchanged by the end of the trial in both groups ($p > 0.05$, Table 2). Comparison of the magnitude of changes between the groups revealed a significant difference for HDL-C ($p < 0.05$). In addition, the extent of serum total cholesterol reduction was greater in the *H. persicum* group, with a borderline significance level ($p = 0.051$). As for the amount of change in LDL-C and triglycerides, no significant difference was observed between the groups ($p > 0.05$, Table 6).

**TABLE 1**
**Clinical and Biochemical Characteristics of *H. persicum* and Atorvastatin Groups**

| Parameter | | *H. persicum* Group | | *n* | *p* Value | Atorvastatin Group | | *n* | *p* Value |
|---|---|---|---|---|---|---|---|---|---|
| | | Pretrial | Post-Trial | | | Pretrial | Post-Trial | | |
| Age (years) | | 52.89 ± 7.81* | — | 18 | — | 57.18 ± 9.00 | — | 34 | — |
| Female (%) | | 45.8 | — | 18 | — | 55.9 | — | 34 | — |
| Cardiovascular disease hospitalization (%) | | 5.6 | — | 18 | — | 0 | — | 34 | — |
| Hypertension (%) | | 27.8 | — | 18 | — | 50.0 | — | 34 | — |
| Diabetes mellitus (%) | | 5.6** | — | 18 | — | 44.1 | — | 34 | — |
| Angina pectoris (%) | | 11.1 | — | 18 | — | 17.6 | — | 34 | — |
| Stroke (%) | | 5.6 | — | 18 | — | 0 | — | 34 | — |
| Smoking (%) | | 11.1 | — | 18 | — | 5.9 | — | 34 | — |
| Family history of | Diabetes mellitus (%) | 27.8** | — | 18 | — | 67.6 | — | 34 | — |
| | Hypertension (%) | 61.1 | — | 18 | — | 61.8 | — | 34 | — |
| | Cardiovascular disease mortality (%) | 33.3 | — | 18 | — | 30.8 | — | 13 | — |
| Weight (kg) | | 75.50 ± 11.73 | 74.78 ± 11.33 | 18 | >0.05 | 75.03 ± 12.70 | 74.80 ± 12.61 | 32 | <0.05 |
| BMI (kg/m$^2$) | | 27.55 ± 4.52 | 27.25 ± 4.07 | 18 | >0.05 | 28.61 ± 4.33 | 28.52 ± 4.27 | 32 | <0.05 |
| ALT (U/L) | | 21.15 ± 6.58 | 22.69 ± 7.22 | 13 | >0.05 | 23.26 ± 8.87 | 26.48 ± 11.67 | 27 | 0.049 |
| AST (U/L) | | 22.00 ± 11.37 | 20.36 ± 6.03 | 14 | >0.05 | 18.93 ± 5.08 | 21.38 ± 6.41 | 29 | 0.013 |
| ALP (U/L) | | 197.23 ± 41.96 | 194.31 ± 48.83 | 13 | >0.05 | 176.33 ± 42.65 | 178.86 ± 53.35 | 21 | >0.05 |
| FBS (mg/dL) | | 99.44 ± 14.09 | 98.59 ± 11.41 | 17 | >0.05 | 129.44 ± 37.84 | 127.59 ± 37.23 | 32 | >0.05 |
| BUN (mg/dL) | | 16.07 ± 3.59 | 15.67 ± 3.31 | 15 | >0.05 | 24.06 ± 8.13 | 24.94 ± 7.37 | 31 | >0.05 |
| Cr (mg/dL) | | 1.06 ± 0.20 | 1.09 ± 0.18 | 13 | >0.05 | 0.93 ± 0.23 | 0.98 ± 0.24 | 27 | >0.05 |

Values are expressed as mean ± SD. BMI: body mass index; ALT: alanine aminotransferase; AST: aspartate aminotransferase; ALP: alkaline phosphatase; FBS: fasting blood sugar; BUN: blood urea nitrogen; Cr: creatinine. Significant difference in baseline values between the groups: **$p < 0.001$; *$p < 0.05$.

Lipid profile changes were also separately analyzed for each gender. In females, there were significant reductions in LDL-C in both groups ($p < 0.01$ and $p < 0.05$ in atorvastatin and *H. persicum* group, respectively). Regarding total cholesterol, there was a significant reduction in the atorvastatin group ($p = 0.001$) and a borderline significant reduction in the *H. persicum* group ($p = 0.05$). HDL-C was only elevated in the atorvastatin group ($p = 0.001$), while there was no significant change in triglycerides in any of the groups ($p > 0.05$, Table 2). Comparison of the magnitude of changes in lipid profile parameters revealed that the extent of reduction in HDL-C was significantly greater in the atorvastatin compared to *H. persicum* group ($p < 0.05$), but there was no significant difference for other lipid profile parameters ($p > 0.05$, Table 6).

**TABLE 2**
**Comparison of Lipid Profile Parameters between *H. persicum* and Atorvastatin Groups**

| Parameter | *H. persicum* Group | | *n* | *p* Value | Atorvastatin Group | | *n* | *p* Value |
|---|---|---|---|---|---|---|---|---|
| | Pretrial | Post-Trial | | | Pretrial | Post-Trial | | |
| **Overall** | | | | | | | | |
| Total cholesterol (mg/dL) | 250.18 ± 30.42 | 191.00 ± 31.77 | 17 | 0.001 | 234.00 ± 33.42 | 202.78 ± 37.21 | 32 | <0.001 |
| Triglycerides (mg/dL) | 169.67 ± 73.44 | 157.00 ± 55.94 | 18 | >0.05 | 183.15 ± 78.54 | 173.45 ± 65.61 | 33 | >0.05 |
| LDL-C (mg/dL) | 167.55 ± 21.61 | 120.21 ± 28.49 | 17 | 0.001 | 165.37 ± 23.36 | 128.29 ± 27.99 | 18 | <0.001 |
| HDL-C (mg/dL) | 53.00 ± 8.79* | 50.67 ± 6.07 | 12 | >0.05 | 48.19 ± 9.31 | 54.00 ± 10.22 | 27 | <0.001 |
| **Males** | | | | | | | | |
| Total cholesterol (mg/dL) | 241.00 ± 18.45 | 188.33 ± 27.03 | 9 | <0.05 | 230.07 ± 28.51 | 209.80 ± 33.90 | 15 | <0.05 |
| Triglycerides (mg/dL) | 163.80 ± 77.79 | 153.60 ± 59.24 | 10 | >0.05 | 190.92 ± 81.72 | 173.21 ± 77.84 | 14 | >0.05 |
| LDL-C (mg/dL) | 164.68 ± 19.10 | 124.78 ± 21.88 | 9 | <0.05 | 156.67 ± 12.49 | 132.05 ± 22.63 | 8 | <0.05 |
| HDL-C (mg/dL) | 51.00 ± 7.97 | 49.40 ± 8.38 | 5 | >0.05 | 44.91 ± 4.04 | 49.27 ± 6.84 | 11 | >0.05 |
| **Females** | | | | | | | | |
| Total cholesterol (mg/dL) | 260.50 ± 38.67 | 194.00 ± 38.12 | 8 | <0.05 | 237.47 ± 37.76 | 196.59 ± 39.88 | 17 | <0.01 |
| Triglycerides (mg/dL) | 177.00 ± 72.15 | 153.60 ± 59.24 | 8 | >0.05 | 177.42 ± 77.84 | 173.63 ± 79.72 | 19 | >0.05 |
| LDL-C (mg/dL) | 170.77 ± 25.04 | 115.08 ± 35.37 | 8 | <0.05 | 173.32 ± 28.07 | 125.28 ± 32.55 | 10 | <0.01 |
| HDL-C (mg/dL) | 54.43 ± 9.68 | 51.57 ± 4.28 | 7 | >0.05 | 50.44 ± 11.22 | 57.25 ± 11.06 | 16 | <0.01 |

LDL-C: low-density lipoprotein cholesterol; HDL-C: high-density lipoprotein cholesterol. *Borderline significant difference between baseline values ($p$ = 0.051).

**TABLE 3**
**Weekly Diet of the *H. persicum* Group at Baseline and During the Trial**

| Frequency of Consumption | Sample Size | Baseline (Times/Week) | | | During the Trial (Times/Week) | | |
|---|---|---|---|---|---|---|---|
| | | <2 | 2–4 | >4 | <2 | 2–4 | >4 |
| Fat (%) | 18 | 11.1 | 66.7 | 22.4 | 55.6 | 44.4 | 0 |
| Carbohydrate (%) | 18 | 0 | 11.1 | 88.9 | 0 | 27.8 | 72.2 |
| Protein (%) | 18 | 5.6 | 22.2 | 72.2 | 11.1 | 66.7 | 22.2 |
| Fruit (%) | 18 | 22.2 | 11.1 | 66.7 | 0 | 22.2 | 77.8 |
| Vegetables (%) | 18 | 22.2 | 16.7 | 61.1 | 5.6 | 22.2 | 72.2 |

**TABLE 4**
**Weekly Diet of the Atorvastatin Group at Baseline and During the Trial**

| Frequency of Consumption | Sample Size | Baseline (Times/Week) | | | During the Trial (Times/Week) | | |
|---|---|---|---|---|---|---|---|
| | | <2 | 2–4 | >4 | <2 | 2–4 | >4 |
| Fat (%) | 24 | 29.4 | 55.9 | 14.4 | 55.9 | 38.2 | 5.9 |
| Carbohydrate (%) | 24 | 0 | 44.1 | 55.9 | 2.9 | 47.1 | 50.0 |
| Protein (%) | 24 | 8.8 | 61.8 | 29.4 | 14.4 | 61.8 | 23.5 |
| Fruit (%) | 24 | 11.8 | 35.3 | 59.2 | 11.8 | 32.4 | 55.9 |
| Vegetables (%) | 24 | 23.5 | 35.3 | 41.2 | 20.6 | 44.1 | 35.3 |

**TABLE 5**
**Magnitude of Change in Clinical and Biochemical Parameters of *H. persicum* and Atorvastatin Groups**

| Parameter | *H. persicum* Group | *n* | Atorvastatin Group | *n* | *p* Value |
|---|---|---|---|---|---|
| Weight (kg) | -0.72 ± 2.24 | 18 | -0.23 ± 0.87 | 32 | >0.05 |
| BMI (kg/m$^2$) | -0.30 ± 1.01 | 18 | -0.09 ± 0.34 | 32 | >0.05 |
| ALT (U/L) | 1.54 ± 5.39 | 13 | 3.22 ± 7.26 | 27 | >0.05 |
| AST (U/L) | -1.64 ± 9.14 | 14 | 2.46 ± 5.65 | 29 | >0.05 |
| ALP (U/L) | -2.92 ± 30.58 | 13 | 2.52 ± 19.41 | 21 | >0.05 |
| FBS (mg/dL) | -1.53 ± 10.45 | 17 | -1.84 ± 18.75 | 32 | >0.05 |
| BUN (mg/dL) | -0.40 ± 2.03 | 15 | 0.87 ± 5.84 | 31 | >0.05 |
| Cr (mg/dL) | 0.03 ± 0.11 | 13 | 0.06 ± 0.17 | 27 | >0.05 |

Values are expressed as mean ± SD. BMI: body mass index; ALT: alanine aminotransferase; AST: aspartate aminotransferase; ALP: alkaline phosphatase; FBS: fasting blood sugar; BUN: blood urea nitrogen; Cr: creatinine.

As for the male population, serum LDL-C and total cholesterol concentrations were significantly reduced in both groups ($p < 0.05$). However, HDL-C and triglycerides were not significantly altered compared to their baseline levels ($p > 0.05$, Table 2). Magnitude of reduction in serum total cholesterol was significantly greater in the *H. persicum* compared to the atorvastatin group ($p < 0.05$). Amount of change in other lipid profile parameters did not reach statistical significance ($p > 0.05$, Table 6).

## Effect of Treatments on Serum Transaminases, ALP, Creatinine, and BUN

A significant elevation in both serum ALT and AST concentrations were observed in the atorvastatin group ($p < 0.05$). In contrast, these transaminases did not show any significant change in the *H. persicum* group ($p > 0.05$). With respect to serum ALP, creatinine, and BUN levels, there was no significant change in any of the groups ($p > 0.05$, Table 1). Finally, the magnitude of changes in none of the aforementioned parameters differed significantly between the groups ($p > 0.05$, Table 5).

**TABLE 6**
**Magnitude of Change in Lipid Profile Parameters of *H. persicum* and Atorvastatin Groups**

| Parameter | *H. persicum* Group | *n* | Atorvastatin Group | *n* | *p* Value |
|---|---|---|---|---|---|
| | | **Overall** | | | |
| Total cholesterol (mg/dL) | -59.18 ± 49.23 | 17 | -31.22 ± 34.01 | 32 | 0.051 |
| Triglycerides (mg/dL) | -12.67 ± 52.18 | 18 | -9.70 ± 45.41 | 33 | >0.05 |
| LDL-C (mg/dL) | -47.34 ± 38.21 | 17 | -37.08 ± 31.04 | 18 | >0.05 |
| HDL-C (mg/dL) | -2.33 ± 6.73 | 12 | 5.81 ± 7.16 | 27 | <0.05 |
| | | **Males** | | | |
| Total cholesterol (mg/dL) | -52.67 ± 36.07 | 9 | -20.27 ± 26.82 | 15 | <0.05 |
| Triglycerides (mg/dL) | -10.20 ± 50.04 | 10 | -17.71 ± 49.47 | 14 | >0.05 |
| LDL-C (mg/dL) | -39.91 ± 28.57 | 9 | -24.63 ± 26.68 | 8 | >0.05 |
| HDL-C (mg/dL) | -1.60 ± 7.86 | 5 | 4.36 ± 6.96 | 11 | >0.05 |
| | | **Females** | | | |
| Total cholesterol (mg/dL) | -66.50 ± 62.74 | 8 | -40.88 ± 37.42 | 17 | >0.05 |
| Triglycerides (mg/dL) | -15.75 ± 58.07 | 8 | -3.78 ± 42.32 | 19 | >0.05 |
| LDL-C (mg/dL) | -55.70 ± 47.46 | 8 | -47.04 ± 31.91 | 10 | >0.05 |
| HDL-C (mg/dL) | -2.86 ± 6.41 | 7 | 6.81 ± 7.35 | 16 | <0.05 |

LDL-C: low-density lipoprotein cholesterol; HDL-C: high-density lipoprotein cholesterol.

## DISCUSSION

The increasing approach toward medicinal herbs as natural, effective, and safe remedies has led to the identification of several medicinal plants and phytochemicals with promising therapeutic potential against a variety of disorders. Nevertheless, clinical trials investigating the therapeutic effectiveness of medicinal plants at pharmacologic doses have not been sufficiently performed. The present study sought to investigate the impact of supplementation with *H. persicum*, a common dietary spice indigenous to Iran, on serum lipid profile parameters in dyslipidemic subjects. To our knowledge, there have been only a few reports on the biological activities of *H. persicum*, with no previous clinical study on this plant.

Based on the obtained results, adjunctive therapy with *H. persicum* extract along with low-dose atorvastatin (10 mg/day) for 8 weeks is associated with significant reductions in serum LDL-C and total cholesterol concentrations. Although comparisons of pre- vs. post-trial values (Table 1) may suggest that these reductions are weaker than those of the atorvastatin group, % reductions and calculated *p* values (Table 2) reveal almost similar effects in both groups. According to previous phytochemical investigations, furanocoumarins and flavonoids are among the constituents of *H. persicum*[20,21,22]. Therefore, the possible hypocholesterolemic activity of the plant may be attributed, at least in part, to the presence of flavonoids, as these phytochemicals have been reported to exert numerous activities, including lipid-lowering effects[23,24,25,26,27]. However, how much of the cholesterol-lowering effect could be ascribed to *H. persicum* extract remains to be clarified and the bioactive ingredients responsible for this effect need to be identified. Moreover, the hypolipidemic effects of adjunctive therapy with *H. persicum* might be augmented at higher doses of the extract, which needs to be evaluated in future studies along with the possible side effects at these higher doses.

In contrast to LDL-C and total cholesterol, combination therapy with *H. persicum* and low-dose atorvastatin was not found to be effective on serum HDL-C levels, while atorvastatin 20 mg/day was associated with a significant elevation of HDL-C. This is a limitation of this assessed combination therapy, as low HDL-C level is a common lipid abnormality that is associated with CAD risk and is classified among the criteria of metabolic syndrome. Hence, an ideal lipid-lowering regimen is expected to address HDL-C levels as well as other lipid profile parameters.

Another finding of the current study was the significant increase in serum AST and ALT (biomarkers of hepatic injury) in the atorvastatin group, while these significant changes were absent in the *H. persicum* group. This effect could be due to the lower dose of statin or direct hepatoprotective activity of the plant extract.

To sum up, the results of the present study indicated that addition of *H. persicum* extract (500 mg daily) to atorvastatin (10 mg daily) for 8 weeks is associated with significant decreases in serum LDL-C and total cholesterol levels. However, unlike the atorvastatin group, no significant effect on serum HDL-C was observed in the *H. persicum* group. Of note, lack of serum ALT and AST elevation was a positive finding in the *H. persicum* group. The current study had a pilot nature and, therefore, a small patient population. Other limitations of the present investigation included lack of blindness as well as exact and systematic assessment of compliance, adverse events, and interactions. In addition, a more careful follow-up would be necessary to reduce the number of drop outs. Finally, the assigning method that was applied here for randomization could be accompanied by bias as the subsequent allocation would be known in advance. Hence, future placebo-controlled trials with larger patient populations are warranted in order to confirm the present findings. Finally, along with lipid profile parameters, the impact of combination therapy with *H. persicum* and low-dose statin on pleiotropic effects of statins should also be considered for a better comparison between the treatments.

## ACKNOWLEDGMENTS

This work was financially supported by a grant from the Baqiyatallah University of Medical Sciences (Tehran, Iran).
